# Fast Panoptic Segmentation with Soft Attention Embeddings

**DOI:** 10.3390/s22030783

**Published:** 2022-01-20

**Authors:** Andra Petrovai, Sergiu Nedevschi

**Affiliations:** Computer Science Department, Technical University of Cluj-Napoca, Memorandumului 28, 400114 Cluj-Napoca, Romania; andra.petrovai@cs.utcluj.ro

**Keywords:** panoptic image segmentation, environment perception, automated driving

## Abstract

Panoptic segmentation provides a rich 2D environment representation by unifying semantic and instance segmentation. Most current state-of-the-art panoptic segmentation methods are built upon two-stage detectors and are not suitable for real-time applications, such as automated driving, due to their high computational complexity. In this work, we introduce a novel, fast and accurate single-stage panoptic segmentation network that employs a shared feature extraction backbone and three network heads for object detection, semantic segmentation, instance-level attention masks. Guided by object detections, our new panoptic segmentation head learns instance specific soft attention masks based on spatial embeddings. The semantic masks for stuff classes and soft instance masks for things classes are pixel-wise coherent and can be easily integrated in a panoptic output. The training and inference pipelines are simplified and no post-processing of the panoptic output is necessary. Benefiting from fast inference speed, the network can be deployed in automated vehicles or robotic applications. We perform extensive experiments on COCO and Cityscapes datasets and obtain competitive results in both accuracy and time. On the Cityscapes dataset we achieve 59.7 panoptic quality with an inference speed of more than 10 FPS on high resolution 1024 × 2048 images.

## 1. Introduction

Panoptic segmentation provides a complex understanding of the environment by performing both pixel level and instance level classification. The formal introduction of the task in [1] and the availability of panoptic segmentation datasets [2,3,4] have facilitated the rapid progress in this complex prediction task. In panoptic segmentation, each pixel receives a semantic label belonging to either *stuff* classes (uncountable elements in the scene such as road, sky, vegetation) or *things* classes (countable elements such as vehicles, pedestrians and cyclists). Moreover, *things* pixels also receive an instance identifier, which enables instance-level understanding.

The task has a broad applicability in many real-world systems that perceive the environment, such as robotics, automated driving [5] or augmented reality. Such systems require accurate predictions but also real-time processing. Most recent approaches [6,7] focus on achieving high accuracy on public benchmarks by designing novel semantic segmentation heads on top of Mask R-CNN [8] instance segmentation network or by designing clever merging heuristics within the network [9]. However, significant progress has been achieved by fast networks, starting from [10], which builds upon a one-stage detector, to [11], where dense bounding boxes are clustered into instance masks using semantic segmentation and finally, the proposal-free method [12], which predicts instance centers and regresses instance center offsets.

In this work, we propose a simple, fast and robust approach that can generate coherent panoptic segmentation with an end-to-end trainable network. We consider that using post-processing steps like clustering of pixels into instances or merging the instance and semantic outputs based on hand-crafted heuristics, which are not incorporated into the network, leads to sub-optimal results. Therefore we design a merging heuristic-free network that can operate almost in real-time. We leverage the opportunity brought by recent works on fully-convolutional one-stage object detectors [13] that are faster and achieve comparable accuracy with two-stage detectors. We build upon the state-of-the-art anchor-free FCOS detector [13] and extend it with a semantic segmentation head and a panoptic segmentation head. Our embedding branch learns a spatial embedding space where instance pixels are pulled together towards the bounding box center. Guided by object detections, these embeddings are used for generating soft attention maps, which attend to *things* segments from the semantic segmentation branch in order to generate instance masks. In our fully convolutional architecture, the semantic and instance-aware soft attention masks are pixel-wise coherent, therefore a simple multiplication operation can yield the final panoptic segmentation result.

We consider that our work has practical importance: the proposed network has a single-stage lightweight architecture, a simplified inference pipeline and does not require merging heuristics for panoptic segmentation. By consequence, the inference speed is high and could be easily deployed in real-world applications such as automated vehicles. To this end, we demonstrate that our network can run in real-time on less powerful GPUs, which can be installed on vehicles. Moreover, an advantage of our method is the simplified training procedure in comparison with that of a two-stage network.

To summarize, our main contributions are the following:

1. We propose a lightweight fully convolutional architecture for panoptic segmentation based on a single-stage object detector which we extend with novel semantic and panoptic heads;
2. We propose a novel panoptic head that predicts instance-specific soft attention masks based on instance center offsets and object detections;
3. The proposed network has simplified inference and training pipelines, does not require merging heuristics for panoptic segmentation, and thus is suitable for real-world applications;
4. We perform extensive experiments on COCO and Cityscapes datasets, and achieve faster inference speeds and better or on-par accuracy compared to existing methods.

## 2. Related Work

The panoptic segmentation task has gained popularity since introduced by Kirillov et al. in [1]. In general, panoptic segmentation methods follow two directions, they are either proposal-based methods, also named top-down approaches or proposal-free methods, known as bottom-up approaches.

**Proposal-Based Methods.** These types of methods solve panoptic segmentation by merging instance predictions and semantic segments. They are built on top of an object detector and use object proposals for generating overlapping instance masks. A post-processing step usually follows, which solves the overlaps and conflicts between semantic and instance predictions. Most works [7,14] employ the two-stage instance segmentation framework Mask R-CNN [8] and extend the shared feature extractor with a lightweight segmentation head. UPSNet [9] introduces a parameter-free panoptic head that employs the semantic and instance logits and is supervised with an explicit panoptic segmentation loss. Seamless segmentation [6] introduces a lightweight DeepLab-inspired segmentation head. AdaptIS [15] takes a point proposal from a semantic segment of a *things* class and generates an instance mask corresponding to that point. EfficientPS [16] proposes a new semantic head that aggregates fine and contextual features, a new Mask R-CNN instance segmentation head and a novel panoptic fusion module and reports state-of-the-art results and faster inference time than two-stage panoptic segmentation methods. Lately, one-shot detectors, such as RetinaNet [17] or FCOS [13], have achieved great progress and even surpassed two-stage detectors on public benchmarks. Motivated by their benefits, FPSNet [10] builds upon RetinaNet [17] and achieves a fast inference speed. DenseBox [11] leverages the idea of reusing discarded dense object proposals by matching them with final object bounding boxes. Ref. [18] extends the single-shot object detector RetinaNet with a semantic segmentation decoder and a pixel offset center prediction branch. Our novel single-shot network achieves competitive results with the state-of-the-art. Moreover, we achieve the lowest inference time, which is very important from a practical point of view. Compared to the above proposal-based approaches, our network does not require instance segmentation predictions, heuristics for instance and semantic segmentation merging, but it optimizes the panoptic output in an end-to-end manner. Our solution is conceptually different from FPSNet [10] which views the entire bounding box area as an attention source and incorporates it in the network as a feature representation. In contrast with [18], we propose the use of soft attention masks based on pixel offsets for instance mask generation, which brings superior results over hard clustering of pixels into instances.

**Proposal-free methods.** The second types of approach have not been so exhaustively studied due to their initial inferior performance compared to proposal-based methods. SSAP [19] models pixel-pair affinities in a hierarchical manner and formulates panoptic segmentation as a graph partition problem. Panoptic DeepLab [12] achieves state-of-the-art results on multiple benchmarks and proposes object center prediction and center offset regression for instance mask detection. Our network bears similarities with these kinds of approaches in the sense that the spatial embedding branch encodes the instance pixel-center distance. However, compared to Panoptic DeepLab, we do not adopt a keypoint representation for the object center and directly use the predicted bounding box center. Moreover, we do not enforce an embedding loss on the center offsets and the spatial embeddings are supervised only by the panoptic segmentation loss. And lastly, we do not make hard assignments based on the predicted offsets to the object centers since this would break the gradient flow inside the network, but we use bounding box predictions to generate soft attention masks. By weighting the semantic segments with the instance-specific soft attention masks, the network is able to directly learn the panoptic output.

## 3. Panoptic Segmentation Network

**Figure 1:**
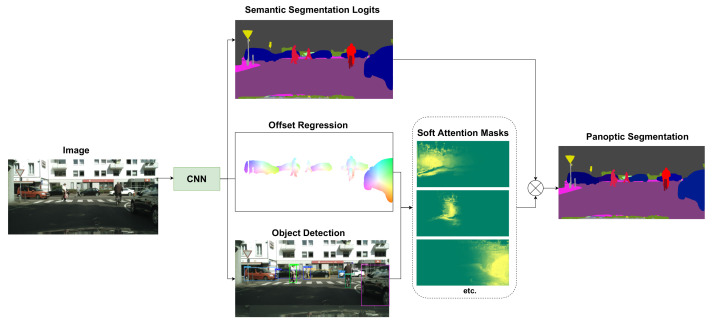
Our fully convolutional network detects objects and predicts semantic segmentation and pixel offsets to instance center. Bounding box predictions, along with pixels offsets, are used to generate instance specific soft attention masks. The panoptic output assembles *stuff* masks and *things* masks scaled by the soft attention maps. The final panoptic segmentation is achieved via per-pixel classification.

**Figure 2:**
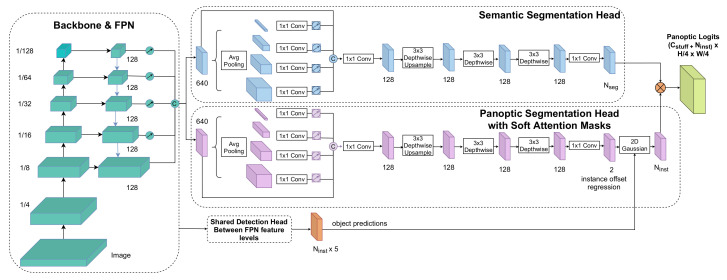
Our network employs a shared backbone and FPN, which we extend with an object detection and classification head, semantic segmentation and panoptic segmentation head. The latter regresses foreground pixels to their corresponding instance centers. The object predictions along with the predicted instance center are used to generate instance-wise soft attention masks that guide the instance mask prediction within the panoptic head by re-weighting semantic segmentation logits. Panoptic segmentation is solved as a dense classification task.

We propose a fully convolutional network for panoptic segmentation, built on top of the anchor-free FCOS detector [13], which we extend with novel semantic segmentation and panoptic segmentation heads. The panoptic head predicts pixel offsets to the instance centers. We do not make hard pixel assignments to the closest center but generate instance-specific soft attention masks that are used to filter instance segments from the semantic segmentation. The panoptic head assembles *stuff* masks and instance masks, and is supervised by the panoptic segmentation loss. In this section, we provide a description of the model architecture and implementation details. We provide a top-level graphical representation of the pipeline in Figure 1 and the detailed architecture of the network in Figure 2.

### 3.1. Model Architecture

**Backbone.** Our network employs a shared backbone for feature extraction and Feature Pyramid Network (FPN) [20] for multi-scale feature representation. We adopt the ResNet50-FPN [21] backbone, where the FPN has a lightweight architecture with 128 channels compared to the default implementation with 256 channels, which is used by other panoptic segmentation networks. We also experiment with the VoVNet2-39 [22] backbone for a boost in accuracy.

**Object detection.** Our instance-specific soft attention masks are guided by object predictions and spatial embeddings. For object detection, we employ the anchor-free, fully convolutional FCOS [13] detector. FCOS solves the object detection problem in a per-pixel prediction manner, thus the network can be easily integrated with other tasks that perform dense pixel classification. This method eliminates the complex computation and hyper-parameters related to anchor boxes, training is easier and inference is faster. The five-scale FPN provides multi-scale feature representation and facilitates multi-scale object detection. Each location in the FPN regresses a bounding box, encoded by a 4D vector representing the distances between the location and the bounding box sides. During training, a location is regarded as a positive sample if it resides inside a bounding box. FCOS assigns objects of different sizes to different levels of the FPN based on pre-defined distances. In order to suppress low-quality boxes predicted by locations far away from the center of the object, the network is extended with a centerness branch. The centerness is defined as the normalized distance between the location and the center of the object. In the Non-Maximimum Suppression (NMS) step, the centerness down weights the scores of low-quality boxes. As a consequence of using the FCOS detector, we inherit the following losses for training the detector: the classification loss (focal loss), the bounding box regression loss (IOU loss) and the centerness loss (binary cross entropy loss). FCOS is accurate and fast and even surpasses some two-stage networks on public benchmarks. Due to its fully convolutional architecture, the network can be further optimized on GPUs using libraries for high-performance deep learning inference, for example [23]. The network avoids the complicated and expensive operations of two-stage detectors and can be easily deployed in real-time vision applications.

**Semantic segmentation.** We propose a lightweight segmentation head that shares the same feature representation with the object detector. Specifically, our semantic head takes the five-level FPN feature maps as input, which are upsampled to 1/8 and then concatenated. We employ the Pyramid Pooling Module (PSP) [24] on top of the concatenated features with 640 channels in order to capture long-range information. We follow the default design of the PSP. First, it applies four parallel average pooling operations, which results in feature maps of sizes: [1×1], [2×2], [3×3] and [6×6]. Next, a [1×1,640] convolution operation and upsampling to 1/8 follow at each pyramid level. The input of the PSP module and its output are concatenated and the multi-scale features are fused with a [1×1,128] convolution. Next, we apply an upsampling stage, which consists of [3×3,128] depthwise separable convolution, Instance Normalization, ReLU activation and 2× bilinear interpolation. To refine the features, we apply another two [3×3,128] depthwise separable convolutions. A final $\left[1\times 1,N_{seg}\right]$ convolution and per-pixel softmax are used to generate the class predictions. In order to supervise the semantic segmentation branch, we minimize the weighted bootstrapped cross entropy loss [12], which applies a larger weight to small sized instances.

**Panoptic Segmentation.** We propose a novel panoptic segmentation head that generates pixel-wise coherent instance-aware soft attention masks and semantic segmentation. Therefore, a simple multiplication operation between the semantic segmentation and instance-aware attention masks can generate the final panoptic segmentation output.

With this work, we propose to unify the training and inference processes and enable the end-to-end learning of panoptic output without requiring any post-processing steps such as clustering or merging between the instance and semantic outputs. We argue that optimizing the final objective is beneficial and results in more accurate predictions. Removing merging operations leads to a faster execution speed and the simplified pipeline overcomes any possible deployment difficulties.

A few works [12,25] formulate instance segmentation as the association of instances’ pixels $P=\{p_{1},p_{2},\ldots ,p_{n}\}$ to the instances’ centroid where $C=\{c_{1},c_{2},\ldots ,c_{k}\}$ is the set of clusters encoded by their instance centers, where *k* is the number of instances in the image. This is accomplished by regressing offsets vectors $O=\{o_{1},o_{2},\ldots ,o_{n}\}$, that represent the distance between a pixel $p_{i}$ and its corresponding instance centroid $c_{k}$, such that $c_{k}=p_{i}+o_{i}$. One approach for identifying instances is to employ a clustering algorithm to group pixels based on their predicted center and their true instance center. The location of the true instances centers are unknown. In order to detect them, Cheng et al. [12] proposes a keypoint representation and Neven et al. [26] introduces an advanced loss function to learn its location based on a seed map.

We formulate instance mask prediction as the problem of predicting offset vectors $c_{k}=p_{i}+o_{i}$ from instance pixels to the instance center. The shared backbone is extended with the panoptic segmentation head, that has the same architecture as the semantic segmentation head as seen in Figure 2. However, we change the final convolution to [1×1,2] such that it outputs the pixel offsets on the *x* and *y* axis. In order to get the instance masks, we do not perform a hard clustering to the nearest center as in previous work. Since most datasets contain both large and small objects, the distance between instance pixels and the instance center may have a large variety of scales. In the case of large objects, learning offsets from pixels that are far away from the center might be more difficult. To alleviate this issue, we propose to relax this constraint by introducing soft attention masks for each instance, computed based on the true instance center, the predicted pixel offsets and the object size.

Specifically, to obtain the soft attention masks, we first need to compute the true instance centers. Given the predicted bounding boxes, we consider the center of the box $c_{k}$ as the true instance center. The object detector outputs a set of bounding boxes $B=\{b_{1},b_{2},\ldots ,b_{k}\}$:

(1) $$b=\left\{\left(x_{1},x_{2},y_{1},y_{2}\right),centerness\right\},s\in \left\{1,N_{things}\right\},$$

where $\left(x_{1},y_{1}\right)$ is the coordinate of the top left corner, $\left(x_{2},y_{2}\right)$ is the coordinate of the bottom right corner and *s* is the semantic class of the object. The centerness value between 0 and 1 is used in the NMS step. Using the top left corner and the bottom right corner coordinates, we compute the true instance center located at $\left(x_{k},y_{k}\right)$.

Secondly, to compute the soft attention masks, pixel offset predictions from the panoptic head are employed. For each location in the image belonging to instances $\left(p_{i},p_{j}\right)$, we predict an instance offset to its center, such that the predicted instance center is $c_{k}=\left(p_{i}+o_{i},p_{j}+o_{j}\right)$.

Thirdly, the object size will be used for computing the soft attention masks. The object size is determined by the height and the width of the bounding box h×w given by the object detector.

We define a soft attention mask $A_{k}$ for each detected object, using an instance-wise probability distribution function that transforms the predicted offsets from the panoptic head into probabilities of pixels belonging to that instance. Specifically, the 2-dimensional Gaussian function, with the mean at the bounding box’s center and the standard deviation given by its size, transforms predicted centers into probabilities:

(2) $$A\left(p_{i},p_{j}\right)=exp\left(-\frac{{\left(p_{i}+o_{i}-x_{k}\right)}^{2}}{w^{2}}-\frac{{\left(p_{j}+o_{j}-y_{k}\right)}^{2}}{h^{2}}\right).$$

**Figure 3:**
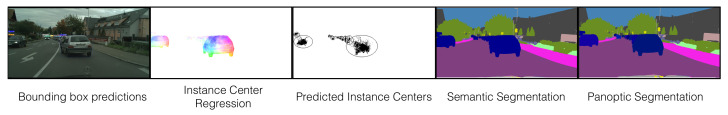
The network performs object detection and outputs bounding boxes and classes for each object. The spatial embedding branch regresses instance center offsets from each foreground pixel. In the instance center regression image, the intensity encodes the magnitude of the offset vector and the color indicates the orientation of the vector. By moving each pixel location with the predicted offsets, we obtain the predicted instance centers. The predicted instance centers are forced to lie within a region centered at the bounding box center with its size defined by the object’s size.

The soft attention masks modeled by the Gaussian function indirectly pull the instance pixels towards the bounding box center and forces them to lie in an elliptical region around the center. The size of this region varies with each prediction and is determined by the size of the bounding box. By adapting the size of the region to the size of the object, we relax the constraint that offset vectors must point exactly at the center, which is especially beneficial for large objects, where far-away locations are more prone to introduce offset errors. However, we enforce that the predicted centers $(p_{i}+o_{i},p_{j}+o_{j}$ must reside inside this area with large probability at the bounding box center $\left(x_{k},y_{k}\right)$, probability which decays towards the edge of the object as the predicted center location deviates from the true center. A depiction of this concept is presented in Figure 3.

**Figure 4:**
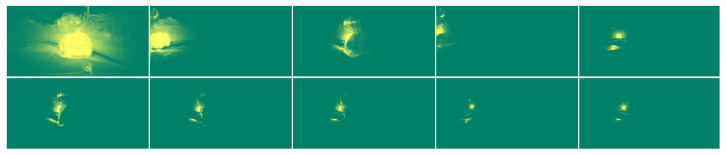
Using the predicted center offsets and the object proposals, we generate soft attention masks for each instance (the original image is in Figure 3). As the saturation of yellow pixels increases, the probability of belonging to the instance is larger. Green pixels have a low probability of belonging to the instance.

As seen in Figure 4, the soft attention masks have large probabilities at the instance pixels and small probabilities otherwise. To obtain the instance masks, we use the soft attention masks as filters on the semantic segmentation. By multiplying an attention mask with the semantic segmentation, we will enhance pixels of that instance by increasing the values and decreasing the logits of background or other instance pixels.

The final panoptic logits $L_{k}$ for instance $C_{k}$ are computed as follows:

(3) $$L_{k}\left(p_{i},p_{j}\right)=S\left(p_{i},p_{j}\right)\left[s_{k}\right]\cdot A_{k}\left(p_{i},p_{j}\right).$$

The semantic class of the box $s_{k}$ is used for selecting the semantic logits *S* of a certain class, which are scaled by the attention map *A*. Panoptic logits will be computed for each detected instance.

Finally, the instance-wise panoptic logits are assembled with the *stuff* logits from semantic segmentation, and we apply softmax over the concatenated logits to minimize the weighted bootstrapped cross entropy loss during training. We note that pixel offsets are supervised only through this loss.

### 3.2. Implementation Details

**Training Setup.** We train our network end-to-end on a system with four Tesla V100 GPUs with a single optimization step. Inference is performed on a single GPU with batch size of 1. In all our experiments, we initialize our backbone with ImageNet [27] pretrained weights [22]. We train and test our model on the Cityscapes [2] and COCO [3] datasets. On Cityscapes, the network is trained with a minibatch of four images for 96 K iterations and decay the initial learning rate with a factor of 0.1 at 76 K and 88 K. On COCO, we train with a batch size of 16 images for 270 K iterations with a step at 210 K and 250 K iterations. We define the loss function as the sum of the object detector losses (bounding box regression loss, object classification loss, centerness loss) and the semantic segmentation loss and panoptic segmentation loss. The loss function is optimized with Stochastic Gradient Descent (SGD) with momentum 0.9, weight decay $1\times 10^{4}$, base learning rate 0.01. Learning rate warm-up is performed for 1500 iterations. During training we apply image augmentations such as random horizontal flipping and scaling with the shorter side randomly chosen from the intervals [768, 1024] for Cityscapes and [640, 800] for COCO.

**Panoptic Segmentation Training.** The panoptic output has the form $\left(N_{stuff}+N_{inst}\right)\times \frac{H}{4}\times \frac{W}{4}$. The first $N_{stuff}$ channels of the panoptic logits represent semantic segmentation logits for stuff classes, while the next $N_{inst}$ channels of the panoptic logits represent instance logits. During training, $N_{inst}$ represents the number of ground truth bounding boxes. In order to generate the soft attention masks, we employ the ground truth bounding boxes and the ground truth object class. During training we must ensure that the order of instance masks in the panoptic output is the same as the order we used when building the panoptic ground truth.

**Panoptic Segmentation Inference.** During inference, the predicted bounding boxes are used to generate the soft attention masks. First, we apply Non-Maxima Suppression (NMS) over the predictions from all FPN levels and select the 100 top scoring boxes with confidence scores higher than 0.3. The panoptic output is built in the same way as in the training phase, but using the filtered object proposals. Softmax is applied over the panoptic logits. The channel of the maximum value gives the semantic class for *stuff* classes and the instance identifier for instances. The semantic class of the instance is given by the class of the predicted object. During evaluation, due to the sensitivity of the PQ metric towards *stuff* predictions [9], we ignore *stuff* masks with areas smaller than 1024 for Cityscapes and 4096 for COCO.

**Pixel Offsets and Bounding Boxes.** Since images are resized to multiple sizes during training, we normalize pixel offsets with the image height in both *x* and *y* directions. We apply the *tanh* activation over the offset predictions, such that they are bounded between [−1,1], which means that a location can regress a maximum offset equal to the height of the image in each direction [26]. Moreover, we normalize the bounding box center and the bounding box size with the height and width of the image.

## 4. Experiments

In this section, we provide experimental results obtained on the Cityscapes and COCO datasets and we discuss ablation studies that led to the current design choices.

### 4.1. Experimental Setup

**Experimental Setup.** Cityscapes [2] is an urban driving dataset with 5000 high-resolution 1024×2048 images. The dataset is split into 2975 training, 500 validation and 1525 test images. Cityscapes provides instance-level annotations for 8 *things* classes and semantic-level annotations for 19 classes.

COCO [3] is a large-scale dataset with common objects having panoptic annotations for 80 *things* classes and 53 *stuff* classes. The dataset has 118 K training images, 5 K and 20 K for validation and testing. All our experiments are done on the *train2017* and *val2017* splits.

**Evaluation Metrics.** We evaluate semantic segmentation with the standard mean Intersection over Union (mIoU). For panoptic segmentation, we adopt Panoptic Quality (PQ), Semantic Quality (SQ) and Recognition Quality (RQ) as defined in [1].

### 4.2. Ablation Studies

In this subsection, we investigate a few architectural design decisions, the influence of different backbones, different loss functions and the importance of the soft attention masks. All our ablation studies are conducted on the Cityscapes dataset and we report inference time for images having 1024×2048 resolution. Our results are presented in Table 1.

**Ablation for Network Backbone.** As seen in Table 2, we perform experiments with multiple backbones. With the VoVNet2-39-FPNlite backbone, we obtain the most accurate results, with 59.7 PQ and 92 ms. FPNlite is a lightweight version of the FPN with 128 channels, whereas the original FPN has 256 channels. We also present our results with the ResNet50-FPNlite backbone. With the ResNet50-FPNlite backbone, we achieve a competitive score of 59.3 PQ and lower inference time of 88 ms.

**Ablation for Network Heads Architecture.** Our panoptic segmentation network is built on the FCOS detector, with which it shares the backbone. We experiment with one vs. two separate heads for semantic and instance offset regression in Table 1. Using a shared decoder network for semantic and instance offset regression yields 58.7 PQ, 75.6 mIoU with an inference speed of 87 ms. Semantic segmentation and instance offset regression may require different feature representations and contextual information, therefore we extend the shared backbone with another decoder. This design change is beneficial to both semantic and panoptic segmentation and increases the PQ score with 1 and mIoU with 0.8. However, it adds an overhead of 5 ms at inference.

In the baseline approach, the semantic segmentation branch classifies pixels into $N_{seg}$ classes, which contain both *things* classes and *stuff* classes. Since the attention masks guide the panoptic segmentation to select instance-level pixels from the segmentation masks, having one category for all *things* classes in semantic segmentation could be enough in theory. Therefore, we experiment with a simplified semantic segmentation head, in which $N_{seg}=N_{stuff}+1$. The network predicts *stuff* pixels and we merge *things* classes into a single category. However, we observe that it is very important to let the network learn every *things* class separately, and obtain 59.7 with multiple classes compared to 57.6 with one category. The attention masks provide rough instance cues for panoptic segmentation and can greatly benefit from using more information about the semantic classes such as background-foreground delimitation and boundaries between different *things* segments.

**Soft Attention Masks Ablation.** We experiment with no attention and two variants of the soft attention masks in Table 1. Our network predicts semantic segmentation and instance center offsets from each foreground pixel. The no attention model performs hard pixel assignments to the instance with the closest center based on the predicted offsets. We build the panoptic segmentation in the same manner and use semantic segmentation logits and bounding box predictions. This baseline network obtains 53.1 PQ. Next, we introduce two types of attention masks. In both approaches, we use a 2-dimensional Gaussian function on top of the instance center regression branch to output the probability of a pixel to belong to an instance. A simplified Gaussian function with standard deviation equal to 1, where the object size is not considered, yields 57.2 PQ. We observe an increase of more than 4 when using soft attention masks compared to the hard clustering counterpart. However, since the dataset contains a variety of objects of different sizes, we find it important to take this into consideration when creating the soft attention masks. By incorporating the scale of the object into the Gaussian function, we relax the constraint that the pixel offsets need to point exactly at the object center and account for possible errors that might happen for far away pixels in large objects. Therefore, we introduce a region defined by the object size around the bounding box center, where the predicted centers must reside. Experiments demonstrate that this design choice is very important and brings a more than 2.5 increase in the panoptic quality score.

**Loss functions Ablation.** Our baseline network is trained with the object detection losses and the panoptic and semantic segmentation losses. All losses are equally weighted in the final loss. We perform multiple loss functions ablation studies in Table 1. First, we investigate the influence of the semantic segmentation loss. By training the network without the semantic segmentation loss we obtain 58.5 PQ and 74.4 mIoU. The network can successfully predict both *stuff* classes and instances by learning with panoptic segmentation loss. However, by adding direct supervision to the semantic segmentation branch, the semantic segmentation is able to provide important cues regarding the boundaries of objects with different classes; therefore, we achieve more accurate panoptic and semantic predictions with 59.7 PQ and 76.4 mIoU. An instance offset regression loss can be introduced on the spatial embedding branch. We employ the L1 loss to pull pixels towards the bounding box center. We do not observe any benefit of adding this extra supervision, but this could be due to the fact that all losses are equally weighted. Further experiments with different loss weights might lead to better results.

### 4.3. Performance on Cityscapes

**Figure 5:**
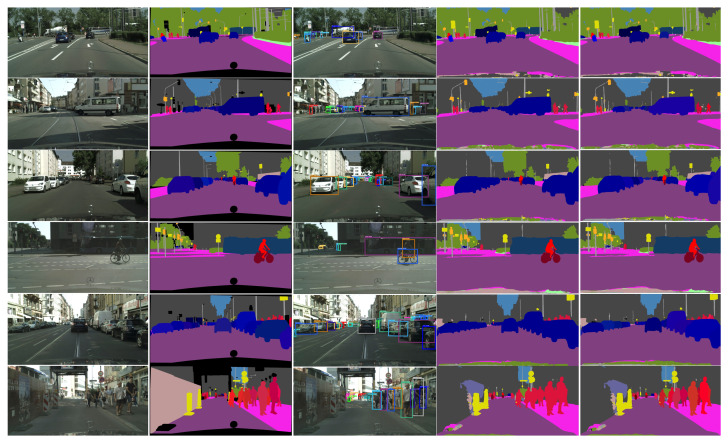
Semantic and panoptic segmentation results on the Cityscapes dataset. From left to right: image, panoptic segmentation ground truth, object detection, semantic segmentation and panoptic segmentation. In the panoptic segmentation the color encodes the semantic class and the instance identifier. Our network can accurately segment objects of various sizes and can handle difficult scenarios with occlusions. Best viewed in color and zoom.

**Panoptic Quality.** In Table 3, we compare the results of panoptic and semantic segmentation on the Cityscapes validation set with other bottom-up and box-based two-stage or single-stage methods. We train only on the fine annotations and no test-time augmentation is used. We report their results obtained with ResNet50 for a fair comparison. Compared to two-stage approaches, our network achieves comparable results with UPSNet [9] and Panoptic-FPN [14] and is outperformed by Seamless Panoptic [6] and EffficientPS [16]. However, our network runs almost twice as fast as most of the two-stage networks. Compared to bottom-up approaches, our network ranks second after Panoptic DeepLab [12]. With a more powerful backbone, VoVNet2-39-FPNlite, we match the accuracy of Panoptic DeepLab. From the single stage methods, our network achieves the best results in terms of both accuracy, surpassing FPSNet [10] with 4.2 PQ and DenseBox [11] with 0.5 PQ. In terms of speed, our method is slightly outperformed by Prototype Panoptic [28] but is more accurate, with a PQ score difference of 2. We provide visual results for panoptic and instance segmentation in Figure 5.

**Runtime.** In Table 3 we report the end-to-end inference time of the networks. The time measures the forward pass of the network on 1024×2048 images and includes the NMS step. The execution time is measured on a Tesla V100 GPU with batch size of 1. Our network with the ResNet50-FPNlite backbone runs the second fastest with 88 ms and using the VoVNet2-39-FPNlite backbone we achieve 92 ms. Obtaining accurate instance segmentation is important, but achieving a good trade-off between accuracy and speed is also important from a practical perspective. We obtain a good trade-off between accuracy and speed because, compared to other approaches, we have a small accuracy drop, but much faster inference speed.

### 4.4. Performance on COCO

**Figure 6:**
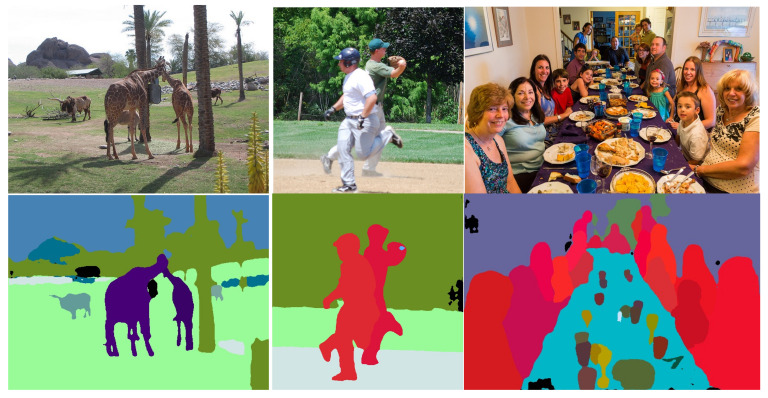
Visual panoptic segmentation results on COCO val dataset. Our network is able to correctly segment partially occluded instances and crowded scenes. In the panoptic segmentation, the color encodes the semantic class and the instance identifier.

We provide a comparison in terms of PQ and inference time with the state-of-the-art on the COCO validation set in Table 4. On COCO, with our ResNet-FPNlite backbone, we achieve 34.4 PQ and 45 ms inference time on images, which are resized such that the shortest side is 800. By downsizing the input image to 640, we obtain a speed-up to 32 ms with a PQ drop of 1. Compared to bottom-up methods, we achieve comparable performance, but smaller inference time. Two-stage approaches [9,14,30] based on Mask R-CNN [8] provide the most accurate segmentation, having the highest PQ scores. At the same time, two-stage methods are the slowest. Compared to UPSNet [9], our network is 2.5× faster. Our network surpasses the single-stage SingleShot [18] network with 2.2 points in PQ with a similar inference time 43 vs. 45 ms. The SingleShot network processes lower resolution input images, so we also downscale the input for a fair comparison. We still achieve higher PQ score 33.4 vs. 32.4 PQ, while being much faster 32 ms vs. 43 ms. Although our segmentation quality for stuff classes is lower (27.1 vs. 28.3), our panoptic segmentation head with soft attention masks brings a significant improvement for things categories, where $PQ_{th}$ is 4.5 points higher. The single-stage DenseBox [11] outperforms our results with a 2.7 PQ margin, at the cost of higher inference time (63 ms vs. 45 ms). We observe that our weakness lies in the segmentation quality for stuff categories. Compared to DenseBox, we have a difference of 4.2 in $PQ_{st}$ for stuff categories. For things categories, our $PQ_{th}$ is comparable (39.3 vs. 41.0), suggesting that our novel panoptic head provides good instance segmentation masks. With a better semantic segmentation quality, we expect both $PQ_{st}$ and $PQ_{th}$ to improve. Our proposed semantic segmentation head is very lightweight and does not handle a very high number of semantic classes (133) well, as in the COCO dataset. To improve the performance of semantic segmentation and increase the panoptic segmentation quality, a solution could be to redesign the semantic segmentation head and increase the capacity of the head by scaling up the width of the convolutional layers. We provide visual results on COCO images in Figure 6.

### 4.5. 2D Panoptic Perception

Our panoptic image segmentation network can be integrated in the 3D perception system of a self-driving car. In our previous work [32,33], the 2D semantic perception solution is a key element of the 3D perception system: a low-level representation of the environment can be built by fusing semantic, instance and geometric information. The goal of the 2D semantic perception system is to detect the road infrastructure, as well as static and dynamic road users. The implementation of such a system can be realized with our panoptic image segmentation network, which provides pixel-level and instance-level classification. A low-level sensor fusion module can associate the panoptic information from images with a 3D point cloud from LiDAR. The augmented 3D point cloud can be further processed to detect and classify 3D objects. Improved detection and classification results can be obtained by processing the enhanced 3D point cloud compared to the simple 3D point cloud. The panoptic segmentation solution can be easily extended to process images from multiple views, in a multi-camera system, in order to provide 360${}^{\circ }$ coverage around the vehicle.

The real-time performance of our panoptic image segmentation network is supported by the new generation of GPU devices and advanced studies in neural network optimization, allowing high inference speed with low computational costs on the new low-power GPUs. To this end, we employ the TensorRT library [23] for high-performance deep learning inference, which performs network optimization and drastically reduces the inference time. Moreover, the TensorRT library generates a high-performance runtime engine which can be easily integrated in C++/CUDA projects and frameworks for automated driving, for example ADTF [34]. We measure the inference time of the optimized panoptic segmentation network on the less powerful NVIDIA GTX 1080 GPU, which can be installed on a self-driving car, as was in our previous work [32]. In Table 5, we provide the results. With the optimized network, we achieve real-time performance on the GTX 1080 GPU, at 32 frames/s, when processing images of 512 × 1024 resolution, without loss in panoptic quality, compared to the unoptimized network. The performance of our network can be further increased by using more powerful GPUs.

## 5. Conclusions

In this work, we propose a novel network for panoptic segmentation. We propose a novel segmentation head and an original panoptic head: object proposals along with the instance center offsets are used for generating instance-specific soft attention maps. The panoptic output guided by the semantic segmentation and the soft attention masks is learned in an end-to-end manner. The proposed network is single-stage, has a simplified inference pipeline, can be easily optimized with deep learning inference engines, which makes deployment on automated vehicles easier. From a practical point of view, we demonstrate that our network runs in real-time on a less powerful GPU, which can be installed on a vehicle, therefore it is suitable for automated driving perception. A good trade-off between accuracy and inference time is achieved on public benchmarks, on both COCO and Cityscapes datasets.

## Figures and Tables

**Table 1:** Ablation study on the Cityscapes *val* set with VoVNet2-39-FPNlite backbone. We train with the following settings: **Dec × 1**: one decoder for both offsets regression and semantic segmentation. **Dec × 2**: two decoders for each task. **Att mean**: attention mask is modeled by a Gaussian with standard deviation equal to 1. **Att scale**: attention mask is modeled by a Gaussian with standard deviation equal to the size of the bounding box. **Seg loss**: use semantic segmentation loss. **Reg loss**: use offset center regression loss. **Th seg**: the classes in semantic segmentation are the *stuff* classes and one class for all the *things* classes. Best results are marked in bold.

Dec × 1	Dec × 2	Att Mean	Att Scale	Seg Loss	Reg Loss	Th Seg	PQ	mIoU	Speed (ms)
	✓			✓		✓	53.1	75.4	92
	✓	✓		✓		✓	57.2	74.9	92
	✓	✓	✓	✓			57.6		91
	✓	✓	✓			✓	58.5	74.4	92
	✓	✓	✓	✓		✓	**59.7**	**76.4**	92
	✓	✓	✓	✓	✓	✓	59.6	76.2	92
✓		✓	✓	✓		✓	58.7	75.6	**87**

**Table 2:** Backbone evaluation on the Cityscapes *val* set. Best results are marked in bold.

Backbone	PQ	${\mathrm{PQ}}_{\mathrm{th}}$	${\mathrm{PQ}}_{\mathrm{st}}$	SQ	RQ	mIoU	Inference Time (ms)
**Full image size: 1024 × 2048**							
ResNet50-FPNlite	59.3	52.8	64.1	80.0	72.7	76.0	88
**VoVNet2-39-FPNlite**	**59.7**	**52.9**	**64.7**	**80.2**	**73.1**	**76.4**	**92**
VoVNet2-57-FPNlite	60.4	53.6	65.4	80.3	73.9	76.8	132
**Half image size: 512 × 1024**							
VoVNet2-39-FPNlite	48.9	41.5	54.3	76.7	61.2	69.8	42

**Table 3:** Comparative study with state-of-the-art two-stage and single-stage panoptic segmentation networks on the Cityscapes *val* dataset. Inference time is measured on one Tesla V100 GPU with batch size of 1. Best results are marked in bold.

Method	Backbone	PQ	${\mathrm{PQ}}_{\mathrm{th}}$	${\mathrm{PQ}}_{\mathrm{st}}$	mIoU	Time (ms)
**Bottom-up**						
DeeperLab [29]	Xception-71	56.5	-	-	-	-
SSAP [19]	ResNet50-FPN	58.4	50.6	-	-	-
AdaptIS [15]	ResNet50	59.0	55.8	61.3	-	-
Panoptic DeepLab [12]	ResNet50	**59.7**	-	-	-	**117**
**Box-based Two-Stage**						
MTN Panoptic [7]	ResNet50-FPN	57.3	53.9	59.7	-	150
Panoptic-FPN [14]	ResNet50-FPN	58.1	52	62.5	75.7	-
UPSNet [9]	ResNet50-FPN	59.3	54.6	62.7	75.2	**140**
Seamless Panoptic [6]	ResNet50-FPN	60.3	56.1	63.3	77.5	150
EfficientPS [16]	ResNet50-FPN	**63.9**	60.7	66.2	79.3	166
**Box-based Single-Stage**						
FPSNet [10]	ResNet50-FPN	55.1	48.3	60.1	-	98
Prototype Panoptic [28]	VoVNet2-39-FPNlite	57.3	50.4	62.4	-	**82**
DenseBox [11]	ResNet50-FPN	58.8	52.1	63.7	77.0	99
**AttentionPS (ours)**	ResNet50-FPNlite	**59.3**	52.8	64.1	76.0	88
**AttentionPS (ours)**	VoVNet2-39-FPNlite	59.7	52.8	64.7	76.4	92

**Table 4:** Comparative study with state-of-the-art two-stage and single-stage panoptic segmentation networks on the COCO *val* dataset. Inference time is measured on one Tesla V100 GPU with batch size of 1. After each method name we place the size of the input to which the shortest side of the image is scaled. Best results are marked in bold.

Method	Backbone	PQ	${\mathrm{PQ}}_{\mathrm{th}}$	${\mathrm{PQ}}_{\mathrm{st}}$	Time (ms)
**Bottom-up**					
DeeperLab [29]	Xception-71	33.8	-	-	92
Panoptic DeepLab [12]	ResNet50	35.1	-	-	**50**
AdaptIS [15]	ResNet50	34.4	50.	29.3	-
PCV [31]	ResNet50	**37.5**	40.7	33.1	-
**Box-based Two-Stage**					
Panoptic-FPN [14]	ResNet50-FPN	41.5	48.3	31.2	-
UPSNet [9]	ResNet50-FPN	42.5	48.6	33.4	**109**
Unifying [30]	ResNet50-FPN	**43.4**	48.6	35.5	-
**Box-based Single-Stage**					
SingleShot-576 [18]	ResNet50-FPN	32.4	34.8	28.6	43
DenseBox-800 [11]	ResNet50-FPN	**37.1**	41.0	31.3	63
**AttentionPS-800 (ours)**	ResNet50-FPNlite	34.4	39.3	27.1	45
**AttentionPS-640 (ours)**	ResNet50-FPNlite	33.4	37.8	26.7	**32**

**Table 5:** Results of the optimized network running on the NVIDIA GTX 1080 GPU and the more powerful NVIDIA Tesla V100. PQ is measured on the Cityscapes *val* set.

Backbone	GPU	Resolution	PQ	Time (ms)
ResNet50-FPNlite	GTX 1080	1024 × 2048	59.3	70
ResNet50-FPNlite	Tesla V100	1024 × 2048	59.3	44
VoVNet2-39-FPNlite	GTX 1080	512 × 1024	48.9	31
VoVNet2-39-FPNlite	Tesla V100	512 × 1024	48.9	20

## Data Availability

Publicly available datasets were analyzed in this study. Cityscapes dataset can be found here: https://www.cityscapes-dataset.com/ (accessed on 14 January 2022). COCO dataset can be found here: https://cocodataset.org/ (accessed on 14 January 2022).
